# Moving in musicking: the evolving pedagogical practice of the artist-facilitator within asylum seeker centers

**DOI:** 10.3389/fpsyg.2023.1177355

**Published:** 2023-06-22

**Authors:** Georgia Nicolaou, Luc Nijs, Peter van Petegem

**Affiliations:** ^1^Royal Conservatoire of Antwerp, AP University College, Antwerp, Belgium; ^2^Research Unit Edubron, Department of Training and Education Sciences, University of Antwerp, Antwerp, Belgium; ^3^Institute of Musicology and the Arts, University of Luxembourg, Belval, Luxembourg

**Keywords:** artist-facilitator, music, movement, children, asylum seekers, participatory workshops, pedagogical practice

## Abstract

The field of community music has been continuously expanding during the last years alongside the need for experienced musicians who can carry out music activities for culturally diverse groups. Based on previous studies, we identified a need for research-based practices for training musicians and music teachers who wish to facilitate community music projects. We believe that it is important to incorporate reflexive practice in order to inform the planning of the workshops, but also to support the needs of the participants. The article examines the evolution of the pedagogical practice of the artist-facilitator in active music making with children, during a series of movement-based musical workshops at an asylum seeker center in Netherlands. We used an exploratory case study integrating Action Research, in order to focus on the artist-facilitator’s pedagogical practice, the participatory role of the children and the content of this type of workshops. The researchers describe the adopted pedagogical approach based on a set of guiding principles and key components that supported the design and content of the workshops. Based on a cyclical process (plan-act-observe-evaluate), the findings from every cycle were incorporated in the next one by analyzing the video footage of the workshops and the immediate reflections of the artist-facilitator. Data analysis revealed a set of recurring themes that reflect crucial aspects of the artist-facilitator’s practice. Furthermore, a set of pedagogical implications are proposed that can be directly implemented within the practice of artists-facilitators who wish to engage in activities with children at asylum seeker centers.

## 1. Introduction

Cultural participation, i.e., the opportunity to contribute and be acknowledged as a valuable participant in cultural interaction (Schuff, 2014), is a right for every individual (United Nations, 1948). A particular group of people who are often deprived from cultural and artistic participation, are refugees who seek asylum and reside at an asylum seeker center. According to the United Nations convention (UNHCR, 2010), a refugee is defined as “someone who is unable or unwilling to return to their country of origin owing to a well-founded fear of being persecuted for reasons of race, religion, nationality, membership of a particular social group, or political opinion”; and an asylum seeker is defined as someone who is seeking international protection (IOM, 2019). Refugee and asylum-seeking children are in a particular situation that often leads to less autonomy due to a lack of (1) independence from controlling influences (liberty) and (2) the possibility or ability to act intentionally (agency; Björn and Björn, 2004). As such, they may be more susceptible to adverse mental health outcomes due to their frequent exposure to trauma and dependency on adults who themselves may be experiencing detrimental effects of trauma (Nielsen et al., 2019; Gandham et al., 2021).

Moreover, asylum-seeking children often face challenges adapting to new cultural norms and practices, which can lead to a sense of cultural dislocation and confusion, especially for those who have experienced trauma or separation from their families (UNICEF, 2016). As such, they might experience a significant shift in their cultural identity. Cultural participation can play a key role in helping children to develop a sense of belonging and connection to both their own cultural identity and the new context. According to Kenny (2016), access to cultural participation in their new context through arts-based activities can be promoted through the creation of “communities of musical practice,” with people from different backgrounds engaging in a creative process of shared music-making. Such communities create a safe space for intercultural or cross-cultural exchange (Crawford, 2020). In addition, they can foster positive change, social integration and wellbeing (Vougioukalou et al., 2019). Considering residents of asylum seeker centers, active music making has proved to work as a catalyst for fostering a sense of belonging, collaborative learning and identity building (Kenny, 2016, 2018). For example, Weston and Lenette (2016) argue that the facilitation of participatory music practices can contribute to the creation of “a cultural and performative space” where asylum seekers can experience a sense of stability and inclusion. For asylum seeking children, music, movement, dance and play within a group can provide significant opportunities for social interaction, relief, emotional expression, and the growth of self-esteem (Marsh, 2017). Nijs and Nicolaou (2021) further argue that the powerful combination of music with movement may contribute significantly to help children develop a flourishing life. Taking into account the fact that the number of refugees that seek asylum among European countries is continuously increasing (UNHCR, 2022), there is a growing interest in socio-artistic practices that promote cultural participation of asylum seekers through community music projects (Kanellopoulou, 2019; Marsh, 2019).

The field of community music practice has been evolving steadily over the past decades, implementing multiple forms of learning, including informal learning, non-formal education, and formal instructional strategies (Higgins and Willingham, 2017). In general, community music interventions are shaped by active music making, incorporating the intentions and aspirations of the participants, and involving participatory teaching and learning practices (Veblen and Waldron, 2012). According to Higgins and Bartleet (2018), it is indeed important to create a dynamic relationship between facilitator and participants through collaboration and a sense of shared purpose. As such, community musicians and music educators are committed to enabling accessible music making for all members of the community (Higgins, 2012), supporting the conviction that cultural participation should be accessible to anyone.

While some Higher Music Education institutions (HMEi’s) integrate programs that prepare musicians to take on a facilitator role in community music projects (e.g., CreArC at Rhythmic Music Conservatory Copenhagen, Community Music MA at University of Limerick, Community Music BA and MA at Wilfrid Laurier University), in most conservatoires or university colleges this is not the case. Given the growth in community music projects, and the need for music education in HMEi’s (i.e., performance, composition, production) to reflect the current societal needs that the future music professionals will have to confront (Lorenzo de Reizabal, 2022), a broader training of musicians and music teachers who are willing to facilitate community projects (Brøske, 2020; Gaunt et al., 2021) is required, for example, to help them effectively carrying out creative workshops inside asylum seeking centers. At the same time, despite these needs and the scholarly interest in community music projects, research-based practices that concentrate on the professional development of the artist-facilitator and on the material that is used in these particular types of interventions are still scarce. Kenny (2022) does highlight the importance of incorporating professional development and reflective practice within community music projects. Moreover, Vougioukalou et al. (2019) emphasize that the facilitators should thoroughly consider the types of music used in this type of interventions in order to support positive change, expression and emancipation among participants. Looking into the complexities that the above issues arise, we concur with Timonen et al. (2020) who argue that there is a need to reinvent the professional development of the music teacher/facilitator in intercultural contexts in light of societal issues such as immigration, democracy, or freedom of cultural expression. They further argue that this professional development should be based on cultivating intercultural qualities and responsiveness in working in unpredictable situations (Timonen et al., 2020). According to Timonen et al., such reinvention entails acquiring new skills and redefining goals, leading to a transformational process within the teachers themselves. Bond (2017) further emphasizes the importance of adopting a culturally responsive education where music educators embrace cultural fluidity and remain flexible in a constantly changing society. Alongside the above views, Barton and Riddle (2021) underline the need for a comprehensive professional development for music teachers that considers the socio-cultural context they are part of and encourages informal forms of musicking where learners make meaningful connections to music making experiences.

To address this crucial topic, we conducted a small-scale exploratory case study integrating Action Research in a Dutch asylum center, that concentrated on the artist-facilitator’s practice and the material that is involved in the musical activities. The starting point for this study was the need to question certain topics such as repertoire vs. composing (Bolden et al., 2021), the role of movement in music learning (Bremmer and Nijs, 2020, 2022), the role of improvisation (Ramón and Chacón-López, 2021) and the participatory design of the workshops (Anderson and Willingham, 2020), whereby the teacher is not the sole controller of the knowledge shared (Barton, 2018). Indeed, it is important to consciously choose and evolve the material that is used in musical workshops in light of supporting the needs of the participants. In this exploratory case study, we focused on the combination of music and movement during musical expression and creation, investigating how we can evolve the pedagogical practice of artists-facilitators that engage in workshops with children within asylum seeker centers. Engaging with music and finding or creating meaning in music can be an empowering experience (Leman, 2016). Moreover, doing so together can positively influence the mutual experience of finding meaning or purpose in life (Croom, 2014). Due to the intrinsic link between music and movement (see for example Sievers et al., 2013), engaging with music through movement intensifies the empowering nature of musical interactions (Nijs and Nicolaou, 2021). Based on this link we designed a pedagogical framework for a series of workshops for children. Here, music, movement, improvisation and collective composition merge into a palette of creative tools for children to actively engage in participatory activities.

## 2. Context

### 2.1. Setting

The Asylum seeker centers (AZCs) in the Netherlands are typically open centers, allowing mobility for all residents. Children are obliged to attend school and adults can work under specific conditions (Geuijen et al., 2020). There are several non-governmental organizations that offer creative activities for the residents of the asylum centers around the Netherlands. One such organization is Stichting De Vrolijkheid (Non-profit Organization ‘The Happiness’, see: https://vrolijkheid.nl), that offers several artistic workshops, primarily for children and teenagers in all AZCs in the Netherlands. The mission of the organization is to invest in the development and empowerment of the residents through creative and joyful arts-based activities that promote social interaction and positive experiences (Stichting de Vrolijkheid, 2018; see also Larruina and Ghorashi, 2016).

The setting of the activities offered by the organization may change regularly, since the asylum center is a transition point. People that seek refuge in the Netherlands are temporarily living at the residency until their status is approved. Then, they are offered a house where they can start building a new life. However, for some residents the stay at the refugee center can be longer than for others, due to approval procedures.

The workshops of this project took place in the recreational room of De Vrolijkheid at the Asylum Seeker Center (AZC) Rijswijk in The Netherlands. Participation was on a voluntary basis. Eleven children, six boys and five girls (aged 6 to 11 years old) chose to participate. Their countries of birth were Eritrea (3), Iran (1), Russia (2), Syria (2), and Turkey (3). Four of them communicated in Dutch and seven in English. All the children resided at the asylum center with their families. The young residents of the asylum center are regularly meeting each other during the creative activities of the organization, as well as in the yard of the center. They are usually playing together outside and, when there is a workshop from ‘De Vrolijkheid’, they run from house to house to announce that the activity is starting soon. They are not obliged to stay for the whole duration of a creative workshop, being free to enter and leave whenever they feel like. Yet, it is up to the facilitator to create boundaries that can be respected.

### 2.2. Methodology

The series of workshops at the AZC Rijswijk were organized in the context of an exploratory case study integrating Action Research, in an effort to explore the artist-facilitator’s pedagogical approach in relation to the active involvement of the children in the co-creation of the activities. The exploratory role of case study research involves a flexible approach (i.e., multiple data sources and methods) of collecting data to acquire a thorough understanding of the subjects of interest (Yin, 2003; Alvesson and Sköldberg, 2017).

The methodology followed the Action Research (AR) model. Action Research is considered as having a value in itself, because the process of carrying out the study may effectively alter practice (Cain, 2008). It is usually associated with small-scale research projects with professionals who aim to accomplish a meaningful change in their practice (Odam, 2001). According to Jacobs (2018); Action Research utilizes an epistemology that involves self-critique, reflexive practice and a perspective for self-change. In our case, we based the case study on a cyclical process (plan-act-observe-evaluate; Kelly, 2005; Migchelbrink, 2016), whereby the findings were incorporated from one cycle to the next by (1) analyzing the video footage of the workshops, and (2) an immediate written reflection after each workshop. This research approach served the twofold role of the artist-facilitator, in analyzing and applying the results directly in her professional practice. Here, the artist-facilitator was the first author. She is a composer, pianist, singer, music educator, and an experienced mover. Taking on the role of artist-facilitator and researcher provided the opportunity to take on an insider’s view (Frederiksen et al., 2021) and conduct practice-based research, in which the artist-facilitator and her practice is at the center of the enquiry (Odam, 2001).

The asylum seeker center (AZC) Rijswijk was chosen in agreement with the Non-profit organization De Vrolijkheid, taking into account the schedule availability and the needs for creative activities during the specific months. Posters were placed 2 weeks before the beginning of the workshops at the main entrance of the center and the door of the recreational room used by the organization. The children were also informed about the workshops from the coordinators of the organization during other activities. On the day of the first workshop the artist-facilitator, together with the coordinator, visited the parents and guardians of the children in their apartments in order to inform them about the start of the new workshops and to receive their written consent. An assent form with pictorial figures was given to the children, offering them the choice to express their agreement to participate in a non-verbal way (Arnott et al., 2020).

Data was collected during a series of participatory workshops of 1 h and a half to 2 h between February and March in 2022. Due to practical issues (the center moving to another location), four workshops could be organized. A first data set, which was also the main source of data, was the video footage of the workshops, recorded with a free-standing camera placed in the corner of the room. Every workshop was recorded entirely from the moment that the children entered until the moment they left the room. A second data set consisted of a diary, containing the immediate reflections by the artist-facilitator after every workshop. These reflections were voice recorded and then transcribed verbatim in a logbook by the facilitator-researcher. A third data set encompassed the artist-facilitator’s reflections based on Video Stimulated Recall (VSR). VSR is an adequate method to observe and evaluate teaching practices such as pedagogical thinking, decision making, classroom behavior and professional experience (Gazdag et al., 2019). In our case, VSR offered the possibility to complement the lived experience during the workshop and the immediate reflections in a diary, with a perspective that allows taking some distance from the subjective interpretation (Martinelle, 2020). In addition, the artist-facilitator had the chance to reflect deliberately on herself and her practice (Geiger et al., 2016), and to directly implement the reflections from one cycle to the next during the process of the AR cycle. Finally, at the end of the final workshop, the children were invited to create their own drawings based on their experience during the series of workshops. They were encouraged to visually interpret any thoughts and emotions related to the music and movement workshops. When the drawings were finished, they were asked to explain in words what they had drawn on paper. This was a very fruitful process since the feedback from the children was very rich in terms of images and stories (for a similar approach, see Johnson et al., 2012; Fortuna and Nijs, 2020). Next to the experiences from the workshops, the children’s interpretations (drawings and verbal explanations) included thoughts related to their homeland, their family and their connection to others. The drawings are not considered as a separate data set due to the fact that the activity was carried out once and not systematically. However, the interpretations from the children on their own drawings are included in the video footage and were analyzed through the VSR.

The analysis was twofold. First, during the course of four workshops, the findings from the video analysis and the immediate reflections were incorporated into the next cycle in order to inform the structural changes and the framework of activities for every new workshop. Second, at the end of the series, the researchers analyzed all videos, the immediate reflections and the VSR data using Thematic Analysis, i.e., searching for emerging themes that could reflect the different aspects of the artist-facilitator’s pedagogical practice. Thematic Analysis is a foundational method for qualitative analysis that is used to identify, analyze, and interpret patterns of meaning (themes) across data (Braun and Clarke, 2006). It enables the researcher to perceive and comprehend collective or shared meanings and experiences, while it can be applied among a range of theoretical frameworks (Braun and Clarke, 2012, 2013). The analysis was first conducted by the first author, whose insider’s insights were considered an important asset in the analysis. Acknowledging however that being both researcher and facilitator may have shaped perspectives and introduced potential biases, and recognizing the need for reflexivity to mitigate these influences, data analysis was discussed with the second author (supervisor), and an additional critical friend (researcher with experience on the social impact of music making).

## 3. Pedagogical approach

### 3.1. Rationale of the innovation

The aim of this project was to gain a deeper understanding of the artist-facilitator’s pedagogical practice in movement-based music making with children. Despite the growing body of knowledge on the embodied nature of human interaction with music (see, e.g., Leman, 2016), the importance of such knowledge for music learning (e.g., Bremmer and Nijs, 2022) and the potential of combining music and movement to support well-being (Nijs and Nicolaou, 2021), the integration of music and movement activities is rarely implemented in community music projects. As such, few studies have investigated the professional development of the facilitator within this particular pedagogical practice. Yet, given the increasing number and demand for community music projects, it is important to address innovative approaches in view of professionalizing musicians who wish to engage in the domain of social music making. Furthermore, the focus was on the act of music making together with the children, at that moment of time; no prior knowledge in a musical instrument was needed and no specific output was expected. Regarding the content of the workshops, the approach was based on the collective process of creation, which included music, movement, improvisation and composition.

### 3.2. Pedagogical principles

In this project the artist-facilitator initially designed a series of activities according to a set of pedagogical principles. These principles provided the general framework of the pedagogical approach, allowing an emergent design throughout the project, based on the progression of the action research cycles (plan-act-observe-evaluate).

The *key* principle was adopting a child-centered approach. Since the case study aimed for a participatory role of the children, it was necessary to listen and incorporate their voice during the planning for every workshop. Besides the informed planning through the first step analysis of every workshop, activities focused on creating music together with the children. The use of existing music was limited to the warming up activities, providing a background to support moving. Considering the multicultural background of the children, the chosen music was always instrumental (to avoid lyrics) and non-culturally specific (fusion). All other activities invited the children to experiment with sound produced with their own voice, body and the small pitched (e.g., push bells, metallophones, chime bars) and unpitched (e.g., egg shakers, wooden sticks, maracas) percussion handed to them. In addition, the artist-facilitator provided pre-composed ideas out of which children could choose and then collectively manipulate them in view of composing their own song. This approach supported the idea of collective creativity being a powerful means to collaborate and make music in a group (Ramón and Chacón-López, 2021).

Next, to child-centeredness as a key principle, the pedagogical approach followed a set of *guiding* principles as outlined by Nijs and Nicolaou (2021). A first guiding principle concerns *connecting* to the children that are invited to engage in the activities. This involves connecting to the children’s social and cultural context, by welcoming their cultural background (e.g., songs, stories), musical interests (e.g., different genres) and creative aspirations (e.g., relating to other artforms, creating original music). It also involves engaging in a learning process that fosters collaboration, and participatory sense-making, by working together towards the common goal of creating and performing music in a group.

A second guiding principle concerns *exploring* individual expression through experimentation with expressive alignment of music and movement. This involves improvising freely with movement and sound in activities that are not necessarily bound to rhythmic or melodic structure, to idiomatic movements (e.g., stepping patterns, standing moves) or to rigid structures (e.g., standard form of a song, 4/4 measure). Once children are enabled to connect to embodied experiences, they can be invited to go beyond the comfort zone of the familiar and to engage in an environment that encourages change.

A third guiding principle concerns *blending*, whereby traditional boundaries between for example genres (western canon vs. world music), ages, or activities (respond vs. create, performing vs. composing) are continuously crossed or even blurred to enrich children’s musical experiences. The basis for this principle is the idea that music is an emergent phenomenon rather than something pregiven or fixed.

A fourth guiding principle concerns *cooperating*, through which children engage in a process of working together towards a common goal. The social interaction involved in cooperation promotes peer learning, inviting individuals to share and elaborate on different perspectives.

The fifth and final guiding principle is *sharing* one’s experiences, gathered through the process, which can be seen at two levels. A first level concerns the sharing ideas between the participating children in view of focusing on the same content. A second level concerns sharing one’s experience with the broader community, such as family, friends, peers, teachers or caregivers. Children gain meaning and importance in what they do through presenting their work to the outer world.

### 3.3. Key components

Starting from the pedagogical principles, specific musical actions were chosen as the components that shape an activity as a whole. These musical actions allowed children to participate actively and express themselves in a variety of ways. Most activities combined different musical actions (“blending”), involved working together (“cooperating”) towards a common goal (“sharing”). Moreover, each of the musical actions allowed children to “explore” different ways of expressing oneself through music. Finally, attending the workshops and participating in the different activities together with children from different backgrounds and age groups, but also discovering new ways of interacting with and through music promoted “connecting.”

#### 3.3.1. Music and movement

The aim was to implement the empowering nature of expressing oneself through music and movement during the process of joint creation and musical sense-making (Leman, 2016; Yanko and Yap, 2020). Bodily involvement with music shapes the way we perceive, feel, experience, and understand music (Leman et al., 2018). Engaging together in music and movement activities fosters participatory sense making (Schiavio and De Jaegher, 2017).

For example, during activities that included improvisation, the children were invited to improvise a sound accompanied by a movement or a movement that produced a sound. In this way, they could directly conceive that the body is always involved in sound making, varying from the most subtle arm movement needed to play a small percussion instrument, to the stamping and jumping while performing a body percussion phrase. Moreover, some activities invited the children to choose between being a “sound maker” or a “mover” according to their preference at that particular moment. For instance, during an activity during which they were invited to play with the pentatonic scale, the “mover” was jumping the steps of the scale while the “sound maker” was playing the push bells following the mover. This offered the possibility to support each other and work together complementing each other’s role.

#### 3.3.2. Improvisation

Another major element of the workshops was improvisation. This spontaneous act of expression was the starting point of many activities because it serves the purpose of free experimentation with sound and movement (Diaz Abrahan et al., 2022). For example, children were invited to move freely in space during a warming up activity with colored fabrics, they experimented freely with pitched and unpitched percussion instruments and their voice. Younger children were often given simpler instruments and were supported by the artist-facilitator during improvising. During the process of gathering material for a collective composition, they drew their thoughts freely on paper. In addition, conducted improvisation was also used in several activities, such as guiding the order of those making music and movement, controlling the dynamics, as well as the tempo. Improvisation offered children the possibility of limitless experimentation and the freedom to explore beyond their comfort zone, yet within the safety of the group.

#### 3.3.3. Composition

Composition has a leading role in music education as a form of musical expression, often assembling original elements in the form of songwriting (Hess, 2019). In our case, composition was envisioned as the gathering and organizing of improvised ideas into a certain type of form. If the ideas were captured on paper, recorded or well memorized, they could then be reproduced. The documentation of the form and the detail of capturing music or movement could be chosen by the composers according to the purpose of the activity. Children were the composers and they were assisted by the artist-facilitator who introduced ways of capturing their ideas. One idea was the creation of graphic scores, where they had the freedom to colorfully draw gestures that could be read as music, movement or both. Another way was instant composing through memorizing a collectively composed musical piece, body percussion or movement sequence, which was then taught to the rest of the group in order to perform it in unison. A song that was collectively composed was also performed for the residents of the asylum center that were sitting outside. This performance was suggested by the artist-facilitator and agreed upon by the children who also chose the location within the premises of the asylum center. By composing new pieces of music and movement children experienced the value working towards a shared purpose, but also the significance of owning their own unique material which could be documented and shared with others.

## 4. Results and discussion

The triangulation of the video, immediate reflection data and VSR data allowed reflecting on the approach, identifying recurring elements and grouping the elements into overarching themes, and different subthemes (see Table 1).

**Table 1:** Themes and subthemes retrieved from the data analysis.

Mode of communication	Class management	Engagement	Differentiation
Verbal vs. non verbal	Problem solving	Active participation	Assignment of roles
Instruction vs. collaborative language	Adaptation	Physical presence	Non-linear approach
Artistic vs. didactic approach	Dynamics	Collaboration	Constraints (individual-task-environment)
			Hooks-triggers

Data analysis revealed four overarching themes and different (sub)themes: *Mode of Communication* (Verbal vs. Non-verbal, Instruction vs. Collaborative language, Artistic vs. Didactic approach); *Class Management* (Problem Solving, Adaptation, Dynamics); *Engagement* (Active Participation, Physical Presence, Collaboration) and *Differentiation* (Assignment of Roles, Non-linear Approach, Constraints, Hooks/Triggers). In what follows, we zoom in on the three (sub)themes that represent areas where improvements were deemed to have a significant impact on the artist-facilitator’s pedagogical practice.

### 4.1. Mode of communication: verbal vs. non-verbal

From the video analysis, the immediate reflections and the VSR data, it became clear that the use of verbal language did not always appear to be effective. Often, due to the length of the facilitator’s explanation and to the necessity of translating instructions in two languages (Dutch and English), children seemed to regularly lose their attention. Consider the following excerpt from the first step analysis of the videos of the first workshop by the artist-facilitator:

I noticed that at the beginning of the workshops I was talking more in an attempt to explain every activity in both English and Dutch. Until I finished my sentence, half of the children were running in the room, unable to concentrate. […] For the next session I have to talk less and show more with my body.

The role of language in music instruction is prominent in explaining particular actions and activities (Barten, 1998), especially when used figuratively (Sakadolskis, 2003) or metaphorically (Schippers, 2006). However, in the case of community music in asylum centers, where not all the children speak the same language, non-verbal communication may more effectively support the dynamic relation of artist-facilitator and participants, allowing to bypass the multi-linguistic explanations. This implies for practice that the facilitator should continuously adapt to the situation in order to explain the activities as short and clearly as possible, maintaining children’s attention span.

After the first workshop, the artist-facilitator replaced the verbal explanations that could be effectively evoked through non-verbal language with vivid gestures, in order to enhance clarity and efficiency in instruction (i.e., a gesture for standing in a circle). The following excerpt from the video analysis exemplifies the use of a gesture to show the formation of a circle in space:

I could just use body language instead. […] When we came back inside I did not talk, I just showed the form of the circle with my hands and I tried to do it really vividly. We actually formed a circle faster than before.

Here, the artist-facilitator observed that the action of instructing the children to stand in a circle had been accomplished with the use of a set of gestures. Indeed, research by Schiavio et al. (2018) showed that non-verbal communication sharpened the attentiveness of the participants (young people with a refugee background) in their study when facilitators kept their verbal instructions as short as possible. By relying more on non-verbal communication, artist-facilitators may be able to create a more immersive and stimulating environment that encourages the participants to focus on the activity, overcoming the gap between the verbal instruction and the learner’s understanding (Foletto, 2018). Moreover, Bremmer and Nijs (2020) discuss the role of the teacher’s gestures, emphasizing not only their potential to serve as visual representations that add to the musical, expressive and technical information being conveyed, but also to serve as an attentional anchor to engage learners’ attention. They further explain that pedagogical gestures can appear in multiple forms (e.g., physiological gestures, musical beats gestures) while the body of the facilitator can flexibly switch between modeling, guiding and assessing the learning process (Table 2).

**Table 2:** Activities and participation per workshop.

Theme of workshop	Activities	Participation (number of children)
Connecting Music and Movement	1. Walk in space	11
	2. Say your name with an original movement (standing in circle)	11
	3. Sound carousel	11
	4. Stepping on beat and playing percussion instruments	11
	5. Wooden sticks	11
	6. Moving to music	4
	7. Song with body percussion	4
From movement quality to sound and Graphic Scores	1. Move in space with a colored fabric (with existing music)	10
	2. The body machine in (standing in circle)	9
	3. Put your machine together (in duos and then with the whole group)	8
	4. Sound gesture on paper leading to graphic scores	6
Singing Pentatonic and Playing on pitched percussion	1. Warm up: move in space and freeze with dum	8
	2. Sing with colored push bells	7
	3. Creation of a song with the pentatonic scale	5
	4. Arrangement of song and sharing with others	5
Singing—Connecting—Accompanying with pitched and unpitched percussion	1. Warm-up: move in space with a colored fabric	11
	2. Warm-up: statues with music that stops	11
	3. Claves and rhythm: improvisation within structure	9
	4. Colored push bells and pentatonic scale	8
	5. Creating song with pentatonic scale	8
	6. Drawings: reflecting experiences throughout the music workshops	10

### 4.2. Engagement: active participation

Engagement through active participation (i.e., engagement of students in music-making activities that involve them in creating, performing, responding to, and connecting with music) was meaningful for building a relationship between the artist-facilitator and the participants towards the common goal of experiencing music and movement together. Focusing on active participation appealed to a sense of belonging to the group as developed through their joint participation in other activities such as going to school, participation in other creative activities, or playing together outside. This project responded to a need—as expressed by the asylum center—to offer creative music making workshops that were not available to children above the age of six during that period. This excerpt from the artist-facilitator’s immediate reflections gives an insight into the relationship between children.

Everyone knows each other very well. They all play together every day and they live in a very closed community. They know what everyone does at any moment. […] They follow all the other activities of ‘de Vrolijkheid’ so they are used to being together.

Although the above observation was expected to reflect the children’s active and collective participation, in reality this was not the case during some activities. Based on the analysis of the immediate reflections, it became clear that the artist-facilitator showed concern on the level of active participation which appeared to be an important prerequisite for a successful session. Consider the following excerpt:

When one kid leaves, others follow. It is a big challenge to keep them engaged […]. They went out to play for the break and most of them wouldn’t come back because it was sunny […]. When I managed to bring most of them back, only four children actively participated but at least whatever we did was fruitful.

On the other hand, during the activities that achieved active participation children appeared to enjoy the process of singing and moving together, as shown in the following excerpt from the video analysis.

Collective singing works well—unisono is a good way to start and it gives a sense of support […]. Combination of movement while singing stimulates their excitement.

Singing in unison while moving together proved to increase the level of energy and therefore stimulate active participation. This resonates with earlier findings by Temmerman (2000), who observed that children benefit most musically when they actively participate in a variety of activities as a whole. In our case, collective tasks worked better than individual tasks because children acted in unison in a group they already knew and trusted, creating a sense of unity and shared experience. Indeed, their preference in singing and moving together, shows that deliberate joint action is meaningful under the group formation framework, a theoretical model used to explain how individuals come together to form groups (Cross et al., 2019). Moreover, Higgins and Bartleet (2018) highlight that community music practice should intentionally promote and expand active participation, by enabling accessible music-making opportunities and supporting both individual and group ownership of the music that the participants make.

### 4.3. Differentiation: non-linear approach

The content of the workshops followed a non-linear approach in differentiating the activities, meaning that the plan was adjusted according to the needs of the children at that particular moment of time. Moreover, non-linearity was present in the interaction between the multiple facets of musicking (i.e., composition, improvisation, moving, listening, performing) steering the process of joint creation. Sometimes, spontaneous acts of improvised music making were welcomed, leading to unexpected and beautiful results. Consider this excerpt from the video analysis:

Introducing something new was stimulating […]. Lack of structure in activities led to more experimentation with instruments and free play […]. While focusing on some children in order to teach them the song, others were practicing or freely experimenting with the instrument. […] A spontaneous act of playing the bansuri with a child accompanying me was very exciting for them and for me. They were drawing and we [the artist-facilitator and a child] played for them with no plan—it just happened.

Here, we observed that the flexibility of the artist-facilitator in accommodating particular situations is influential in maximizing the opportunities for the participants to engage in creative musicking through promoting multimodal musical engagements (Lewis, 2020). In a non-linear approach, knowledge is acquired as a result of the interactions between the learner and the environment (Crawford, 2014). By creating opportunities for these interactions to occur, the artist-facilitator can create an inclusive environment that encourages participation and collaboration, while giving emphasis on creativity and a focus on the individual (Chow, 2013). Indeed, the ability to be culturally informed and responsive is an important aspect of the pedagogical practice of artists-facilitators involved in community music projects (Abril and Robinson, 2019). Due to the fact that workshops within asylum seeker centers involve participants with diverse backgrounds, age groups and experiences, the needs and interests of the group can change rapidly. By maintaining a reflexive approach and adjusting the plan or changing the direction of a workshop when needed, artists-facilitators can ensure that the activities remain meaningful for all.

## 5. Practical implications

### 5.1. The role of gestures

When the supportive role of movement in joint musical interaction is addressed, it mostly concerns the learner’s movements. However, this study revealed that also the facilitators’ gestures play a supportive role. This resonates with Bremmer and Nijs (2020), who argue that different types of teacher’s bodily engagement (physical modeling, action demonstration, pedagogical gestures and touch) can support music learning, thereby facilitating the learning process in non-verbal ways. Here, it became clear that, in the context of working in a multilingual and multicultural context, it is useful for artist-facilitators to add more dynamicity to their expressive gestures while instructing or taking part in activities. This implication is directly related to the theme of *Verbal* vs. *Non-verbal* as gestures can either support verbal language or replace the language. Indeed, an expressive gesture that is used deliberately can show a lot of information, especially if it is amplified (Flood et al., 2020). By applying a dynamic range in gestures, a vivid interpretation can be equally clear as a spoken phrase.

### 5.2. Variation of activities

Another practical implication for artists-facilitators is the frequent variation of activities to keep children actively engaged and to provide a multiperspectival approach to music. This of course requires a great amount of sensitivity and responsiveness in order to be proactive as a teacher and maintain a continuous flow within the dynamics of a workshop through the manipulation of different elements (or: constraints; Bremmer and Nijs, 2022) of the activity. Such constraints shape activities by defining or proposing boundaries of behavior at the individual (e.g., vary movement possibilities: hands only or whole body), task (e.g., move or draw) and environmental (e.g., vary materials that can be used: fabrics or a ball) level. However, if a set of activities for specific occasions are prepared, they can be used when they are needed. For example, a set of short activities can be prepared for warming up, to act as energizers, to act as body and mind release, or to aid concentration. The activities can be grouped in categories and used when needed. Such preparation can foster reflexive, accepting and unforced interactions between the facilitator and participants that support the explorative environment of a workshop (Howell et al., 2017). This implication is directly related to the *Non-linear Approach* in differentiating the activities involved in a lesson or a workshop according to the needs of the children at that particular moment.

### 5.3. Support children’s expression

A practical implication that artists-facilitators can incorporate in their practice is the skill to assist children when they appear to have difficulties in expressing themselves, introducing new ideas, or improvising. This implication emerged from the theme of Engagement and *Active Participation*. For children that are introverted, an invitation to improvise or propose a musical idea can be confronting, especially if the rest of the group is watching. Therefore, the artist-facilitator can guide a child by giving a quick suggestion close to his/her ear or inviting the child to imitate a sound or movement. Dividing the group in dyads or triads for some time can also assist expression since children can feel more comfortable in experimenting and sharing ideas within a smaller group (Edmund and Keller, 2019).

### 5.4. Engage bodily movement while singing

A final practical implication we propose which is related to the role of the children and connected to the theme of *Active Participation*, is the engagement of the body while singing. Movement can assist expression and aid the feeling of rhythm while singing. Coordinated movement can vary from stepping and swinging to the underlying rhythm, to involving the body while doing Body Percussion. It can also be synchronized, meaning that all children are doing the same movement, or moving the same rhythmical pattern with a different set of movements. Here, synchronicity in rhythm is essential. A rhythmical movement pattern can be first practiced with the whole group and then the singing phrase can be added to it, repeating many times the movement and singing apart, as well as in synchrony. It is important that the person who leads the group is clearly visible (i.e., positioned in a circle) while singing and moving in order for the children to easily follow. Moreover, the facilitator needs to consider the cultural diversity of the group during the choice of movements proposed based on a sense of attentiveness on self-identity and power-negotiation (Phelan, 2008).

## 6. Limitations

The presented work had some limitations concerning practice and research. First, the limited number of workshops affected the flow and progress of the workshops. For example, there was not sufficient time to build a solid relationship between the participants and the artist-facilitator. This contributed to the level of physical as well as active participation of the children, revealing a relation between the number of workshops and the engagement of the participants. Moreover, due to the fact that the existing framework of the non-governmental organization was already set for the creative activities carried out at the asylum center, the number of the children that participated in every workshop was varying. The liberal context of the creative workshops offered by the Non-profit organization contributed to the level of freedom children had in entering and leaving the workshops, asking for more rigorous boundaries from the artist-facilitator, such as making agreements on being on time, not leaving the room before the completion of an activity and notifying the rest of the group if a choice is made to exit the room. This had an impact on the content of the workshops, the termination of certain activities on odd moments and the flow of the workshop with the remaining children. Despite those concerns, we had to acknowledge that the ultimate purpose of the workshops was to create a supporting space for the children to experience music making in a group, following the guidelines of the organization on attendance. Second, the children’s inconsistent workshop attendance had an impact on the data collection.

## 7. Conclusion

The goal of this study was to gain insights on the artist-facilitator’s pedagogical practice when working with asylum seeking children, whereby activities were built on the strong connection between music and movement. To do so, we set up an exploratory case study, organizing workshops at the Rijswijk Asylum Center in the Netherlands. Analysis of the video and the artist-facilitator’s reflections (immediate and VSR) revealed recurrent themes and subthemes, expressing different aspects of the pedagogical practice that evolved through the progression of the action research cycle. It became clear (1) that *non-verbal communication* plays a significant role supporting verbal instructions and in providing an attentional anchor, (2) that movement-based *active participation* supports building a dynamic relationship between artist-facilitator and participants and provoking both a higher level of active participation and a greater sense of togetherness, and (3) that a *non-linear approach* can steer the process of musical creation through a synergetic interaction between the multiple facets of musicking (i.e., composition, improvisation, moving, listening, performing) and supports reflective practice. The emergence of the themes and subthemes clearly indicates the multidimensionality of a facilitator’s role in working with children in asylum seeking centers, emphasizing the role of gestures, variation in activities and bodily engagement in singing.

Despite some of the limitations, both regarding the workshops and the research, findings of this case study reveal interesting avenues for further research that focuses on the artist-facilitators professional growth. One example is the child-centered approach as a key principle of working with children with migrant and low socio-economic backgrounds. Future research may investigate the participatory role of children in designing the content of workshops as well as in research methodology, emphasizing the need for prioritizing their needs and aspirations. Another example is the role of movement in community music projects. Here, future research may focus on the impact of movement-based creative musical activities to support elements of the well-being of asylum seeking children, such as resilience, social inclusion, and community building.

Furthermore, the presented work may inspire existing and future training programs for musicians and social workers who wish to work in similar contexts. Especially learning about the role of bodily movement in relation to a dynamic approach in which activities are varied through a diversity of expression, may contribute in building a collaborative space for community arts-based initiatives that inform each other. A multidisciplinary training of musicians as well as professionals from other artistic disciplines (e.g., dance, theatre, fine arts) may inform the process of designing workshops and developing adequate skills for working in such contexts. Finally, we would like to emphasize the urgency of reforming the formal music education programs in view of professionalizing musicians that are culturally responsive, sensitive and socially engaged citizens.

## Data availability statement

The processed data and the immediate reflections from the artist-facilitator supporting the conclusions of this article will be made available by the authors, without undue reservation.

## Ethics statement

Ethical review and approval was not required for the study on human participants in accordance with the local legislation and institutional requirements. Written informed consent to participate in this study was provided by the participants’ legal guardian/next of kin.

## Author contributions

GN: designed and conducted the study, collected and analyzed the data, and worked on the manuscript. LN: co-designed the study, assisted data analysis and manuscript writing. PP: assisting in writing the manuscript. All authors contributed to the article and approved the submitted version.

## Funding

This work was supported as an impulse research project by the Royal Conservatoire of Antwerp - AP University College and the University of Luxembourg.

## Conflict of interest

The authors declare that the research was conducted in the absence of any commercial or financial relationships that could be construed as a potential conflict of interest.

## Publisher’s note

All claims expressed in this article are solely those of the authors and do not necessarily represent those of their affiliated organizations, or those of the publisher, the editors and the reviewers. Any product that may be evaluated in this article, or claim that may be made by its manufacturer, is not guaranteed or endorsed by the publisher.
